# Biomedical Applications of Gadolinium‐Containing Biomaterials: Not Only MRI Contrast Agent

**DOI:** 10.1002/advs.202501722

**Published:** 2025-04-25

**Authors:** Xingtong Pan, Menglong Hu, Likun Wu, Erfan Wei, Qiyue Zhu, Letian Lv, Xiuyun Xv, Xinyi Dong, Hao Liu, Yunsong Liu

**Affiliations:** ^1^ Department of Prosthodontics Peking University School and Hospital of Stomatology Beijing 100081 China; ^2^ National Center of Stomatology National Clinical Research Center for Oral Diseases National Engineering Research Center of Oral Biomaterials and Digital Medical Devices Beijing Key Laboratory of Digital Stomatology Research Center of Engineering and Technology for Computerized Dentistry Ministry of Health NMPA Key Laboratory for Dental Materials Beijing 100081 China; ^3^ The Central Laboratory Peking University School and Hospital of Stomatology Beijing 100081 China

**Keywords:** anticancer, antimicrobial, bio‐imaging, Gadolinium, osteogenesis

## Abstract

The potential applications of rare earth elements (REEs) in biomedical fields have been intensively investigated. Numerous studies have shown that doping biomaterials with REEs can enhance their properties. Gadolinium (Gd) is a biocompatible REE that holds promise in biomedical applications. This review examines the use of Gd‐doped biomaterials in osteogenic, antimicrobial, anticancer applications, and in bioimaging and bioprobes, as reported in the literature until December 2024. The included studies demonstrate that Gd‐containing biomaterials promote osteogenesis, enhance antimicrobial properties, and perform well in anticancer applications and bioimaging. Taken together, they point to the considerable potential of Gd‐doped biomaterials and thus to avenues for future research.

## Graphical abstract

This paper summarizes the latest applications and potential mechanisms of the rare earth element gadolinium in biomedical fields, in addition to its traditional application as an MRI contrast agent, including osteogenesis, antimicrobial, antitumor, bioimaging, bioprobes, and so on. This review not only gives us a better understanding the application status of gadolinium in biomedical fields, but also provides some insights for the future application of gadolinium‐containing biomaterials in biomedical fields.

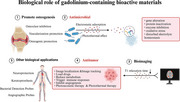

## 1 Introduction

Rare earth elements (REEs) refer to 17 elements in the third subgroup of the periodic table. They include scandium (Sc), yttrium (Y), and the lanthanides, which range from lanthanum (La) to lutetium (Lu). The distinct physical and chemical properties of REEs can be attributed to their unique 4f electron orbitals. REEs have been investigated for their ability to catalyze and activate enzymes, promote cell division, and accelerate tissue healing; they also exhibit antimicrobial activity and good biocompatibility.^[^ ^1^, ^2^, ^3^, ^4^, ^5^, ^6^, ^7^, ^8^ ^]^ The doping of biomedically relevant material with a small amount of a REE can significantly improve its function, which accounts for the use of REEs in tissue regeneration, antibacterial preparations, fluorescent labeling imaging, drug loading, and as antitumor agents.^[^ ^6^, ^9^, ^10^, ^11^, ^12^, ^13^, ^14^, ^15^, ^16^, ^17^, ^18^ ^]^

**Figure 1:**
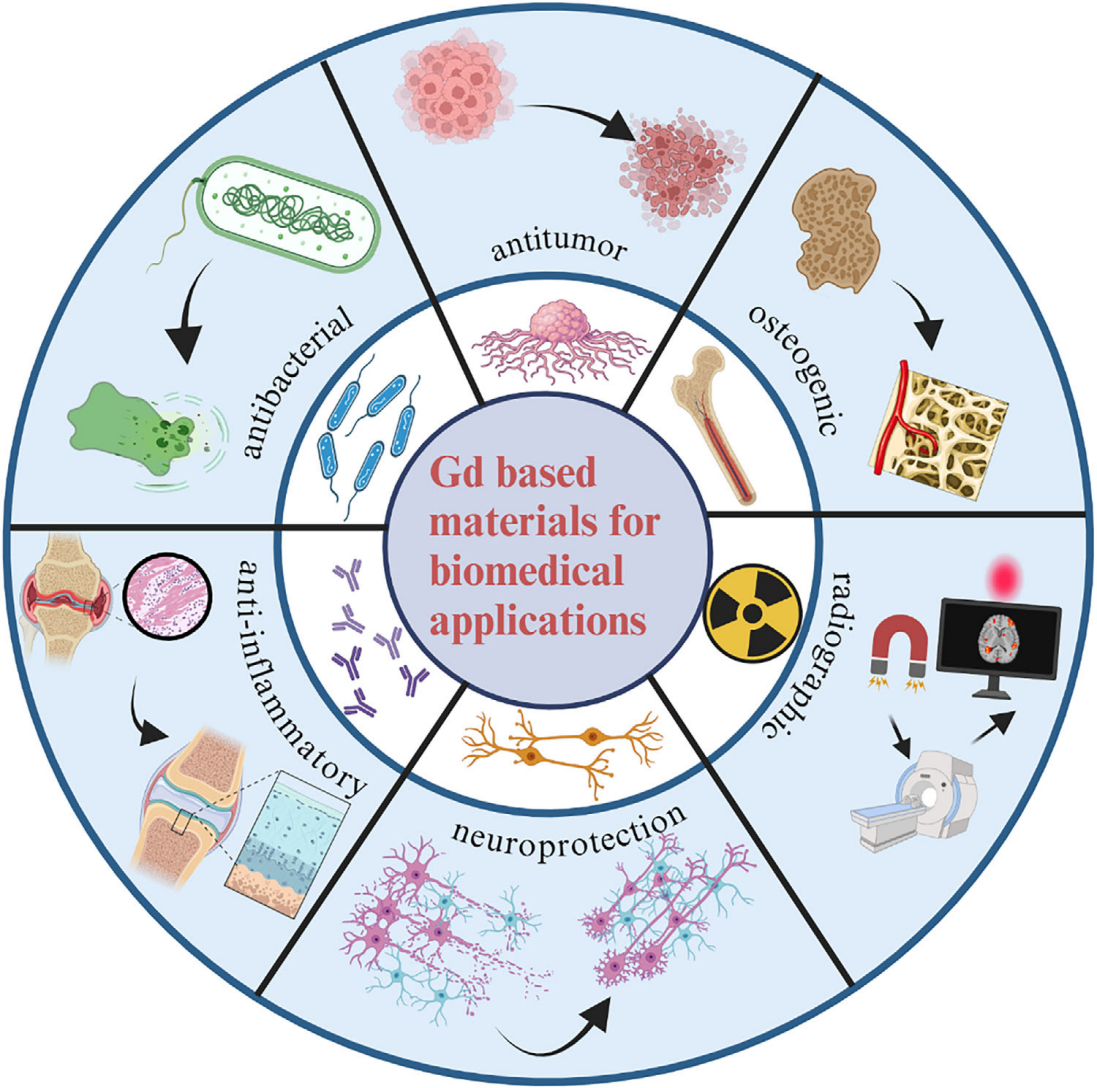
Applications of the rare earth element gadolinium in the direction of biomedical fields. Gadolinium‐containing biological materials play an important role in imaging, antimicrobial, anti‐inflammatory, bone regeneration, antitumor, and even neuroprotection.

Gadolinium (Gd), a group III B lanthanide, is one of the most abundant REEs on the Earth's surface.^[^ ^19^ ^]^ Unlike other REEs, Gd materials are paramagnetic.^[^ ^20^ ^]^ Thus, when used in magnetic resonance imaging (MRI), tissues that accumulate Gd have a much shorter T1 time, resulting in a bright signal on T1‐weighted images.^[^ ^21^ ^]^ Gd‐containing materials also provide a magnetic environment to form noncontact biological scaffolds for use in tissue formation and cell culture.^[^ ^22^, ^23^, ^24^, ^25^, ^26^ ^]^ Gadolinium‐containing materials promote osteogenesis by inhibiting osteoclast proliferation, promoting stem cell differentiation to osteoblasts, and vasculogenesis.^[^ ^27^ ^]^ Similar to other REEs, Gd enhances the antimicrobial properties of encapsulated materials by altering their surface charge and photothermal effects.^[^ ^28^ ^]^ In tumor diagnosis and treatment, gadolinium‐based materials can be used as a contrast agent to visualize the location of tumors, as a carrier to deliver antitumor drugs to the tumor site, disrupt the mitochondrial structure at the corresponding site, inhibit ATP synthesis, and induce oxidative stress to promote the death of tumor cells, thus achieving the multipurpose and highly effective antitumor effect.^[^ ^29^, ^30^, ^31^ ^]^ The utility of gadolinium oxide (Gd2O3) has also been demonstrated, which includes a role in drug delivery, as a diagnostic tool for targeting cancer cells, as a therapeutic drug for cancer treatment, in cell tracking and labeling, and as a biosensor, among other functions (**Figure** **1**).^[^ ^32^, ^33^, ^34^ ^]^

Despite the numerous biomedical applications of Gd‐containing materials, a review of the properties of Gd‐containing materials is lacking. This article summarizes the utility of Gd‐containing materials in osteogenesis, antibacterial, antitumoral agents, and in bioimaging and bioprobes, and in other biological applications, with a view to providing a reference for further research.

## 2 Traditional Applications of Gadolinium—MRI Contrast Agents

The rare earth element gadolinium is the most commonly used contrast agent in MRI. Gadolinium has good paramagnetism, and the unpaired electrons of Gd^3^⁺ generate local magnetic field fluctuations that can enhance the contrast between images of diseased and normal tissues by shortening the longitudinal relaxation time (T1), which leads to signal enhancement in T1‐weighted images (bright signals), and by shortening the transversal relaxation time (T2/T2*) at high concentrations, which interferes with the relaxation of neighboring water protons.^[^ ^35^ ^]^ Gadolinium ions are toxic by themselves, but are less toxic when combined with chelating agents such as DTPA and DOTA to form stable complexes such as Gd‐DTPA. The chelating agent encapsulates Gd^3^⁺ and prevents it from freeing up and causing toxicity, while allowing water molecules to briefly contact gadolinium ions to maintain the paramagnetic effect, and the complexes can be safely metabolized by the kidneys, reducing the risk of toxicity.^[^ ^36^ ^]^ Meanwhile, gadolinium‐based contrast agents have good distribution and pharmacokinetic characteristics; most gadolinium contrast agents are excreted through glomerular filtration and have a short half‐life (≈1.5 h), making them suitable for short‐term imaging needs.^[^ ^37^ ^]^

Due to their biological properties, gadolinium‐containing biomaterials are widely used in neurological imaging, cardiovascular imaging, tumor diagnosis and staging, and abdominal and pelvic imaging.

### 2.1 Gadolinium in Neurologic Imaging

Areas of inflammation, necrosis, edema, and tumor boundaries in the brain cause abnormal enhancement due to disruption of the blood–brain barrier, so gadolinium contrast agent crosses the disrupted blood–brain barrier and enhances the corresponding areas in the T1‐weighted image.^[^ ^38^, ^39^, ^40^ ^]^ Meanwhile, dynamic enhancement scanning (DCE‐MRI) can assess cerebral blood flow and blood–brain barrier permeability.^[^ ^41^ ^]^ Quantification of hemodynamic parameters by rapid intravenous injection of gadolinium contrast agent, using its effect on the local magnetic field (ΔR2 ∗ ΔR2 ∗) to capture the first‐pass effect signal change, is suitable for acute ischemic stroke assessment because of its high temporal resolution.^[^ ^42^ ^]^ Multiple sclerosis (MS) is a chronic incurable disease of the central nervous system (CNS) and a leading cause of neurologic disability in young adults.^[^ ^43^ ^]^ Focal inflammatory and demyelinating lesions scattered throughout the white matter of the brain have long been recognized as a major feature of multiple sclerosis.^[^ ^44^ ^]^ Gadolinium‐enhanced T1‐weighted sequences are sensitive to detect focal demyelinating lesions in the white matter, damage to the blood–brain barrier, as well as tissue loss within the lesion and inflammatory activity to assess disease activity.^[^ ^45^ ^]^ In recent years, it has been found that soft meningeal inflammation plays an important role in disease progression in patients with multiple sclerosis, and that Leptomeningeal Enhancement (LME), which may reflect chronic soft meningeal inflammation, is a potential marker for progressive MS and can be used to assess disease activity and treatment response.^[^ ^46^ ^]^ MRI with field strengths of 3T and above combined with delayed T2 FLAIR sequences, scanned 10 minutes after gadolinium contrast injection, significantly improved the sensitivity of LME detection, and in conjunction with PET‐MRI or optical probes may enhance the ability to dynamically monitor soft meningeal inflammation and guide precise treatment.^[^ ^47^ ^]^

### 2.2 Gadolinium in Imaging of the Cardiovascular System

In addition to vascular imaging of space‐occupying lesions such as cardiac tumors or blood clots, as well as arterial stenosis, aneurysms, or vascular malformations, and assessment of perfusion abnormalities in areas of myocardial ischemia or infarction, gadolinium‐containing contrast agents are important for accurate diagnosis and prognosis of a number of diseases.^[^ ^48^, ^49^ ^]^

Cardiac nodular disease is a multisystem inflammatory disorder associated with elevated risk of ventricular arrhythmias and sudden cardiac death. Late gadolinium enhancement (LGE) serves as a marker of myocardial fibrosis or inflammation, and the use of LGE as a prognostic indicator in cardiac magnetic resonance (CMR) may provide prognostic information.^[^ ^50^ ^]^ Gadolinium‐based contrast agents also play a role in the visualization of areas of myocardial infarction and in the diagnosis and prediction of long‐term outcomes in cardiomyopathies such as dilated and nondilated cardiomyopathies, as well as ischemic cardiomyopathies.^[^ ^51^, ^52^ ^]^ With deep learning, late CMR can now quantify LGE, measure myocardial scarring and microvascular obstruction, and have clinical implications for the diagnosis of patients with acute myocardial infarction.^[^ ^53^ ^]^

### 2.3 Gadolinium Imaging in the Abdomen and Pelvis

Gadolinium has renal excretion properties.^[^ ^37^, ^54^ ^]^ Therefore, by designing gadolinium‐containing contrast agents into small‐sized, well‐dispersed kidney‐cleared nanoparticles for the assessment of glomerular filtration rate (GFR) and renal function abnormalities, the investigators have provided a new strategy for noninvasive and highly sensitive diagnosis of renal dysfunction, which is expected to facilitate early intervention and personalized treatment.^[^ ^55^ ^]^

Conventional assessment of intestinal wall inflammation, including Crohn's disease, has relied on colonoscopy, which is unable to detect extraintestinal lesions and has invasive limitations. Studies have demonstrated that the Magnetic Resonance Enterography (MRE) index (especially the simplified MaRIA) with gadolinium‐based contrast agents is effective in evaluating the response to Crohn's disease treatment, providing a reliable tool for clinical trials and individualized treatment. A reliable tool for clinical trials and individualized therapy.^[^ ^56^ ^]^ Early clinical trials have shown that gadolinium‐enhanced magnetic resonance imaging (G‐MRI) helps to differentiate between Crohn's disease (CD) and ulcerative colitis (UC) in pediatric IBD patients.^[^ ^57^ ^]^

In conclusion, gadolinium‐containing contrast agents enhance the T1 signal through paramagnetism, provide high‐contrast imaging with a high degree of safety, and have the potential for targeted modification, making them an indispensable tool for MRI.

## 3 Gadolinium and Osteogenesis

**Figure 2:**
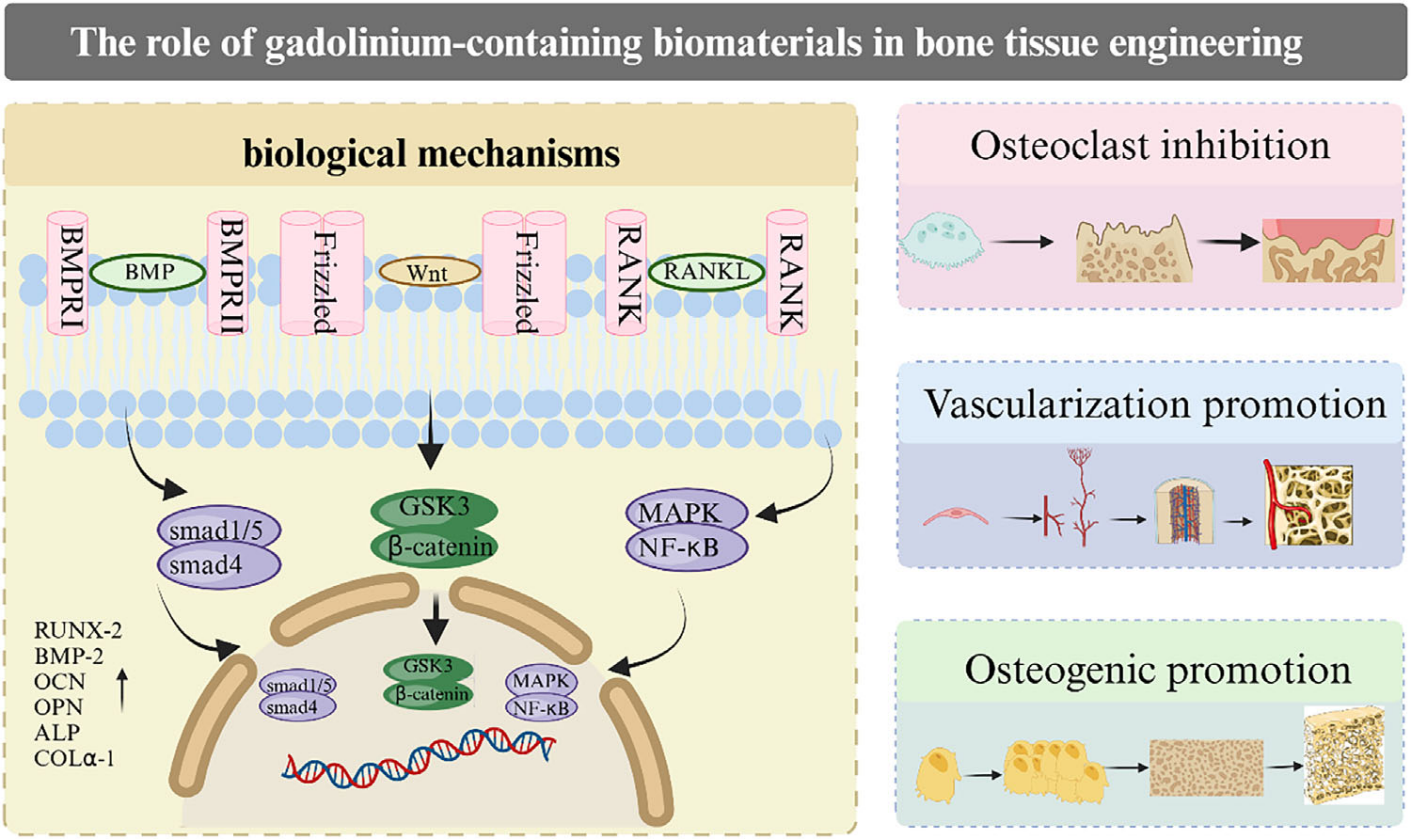
The role of gadolinium‐containing biomaterials in bone tissue engineering. Gadolinium‐containing biological material promotes osteogenic differentiation of bone marrow mesenchymal stem cells, inhibits osteoblastic differentiation and promotes vascularization.

Bone tissue engineering refers to the repair of bone tissue defects by inoculating seed cells on biological scaffolds or implanting mixed growth factors in bone defects.^[^ ^58^, ^59^, ^60^, ^61^ ^]^ The good biocompatibility of Gd elements has been demonstrated in many recent studies, in which Gd‐containing biomaterials were used to promote osteogenesis in vivo and in vitro by combining them with other REEs, biologically active materials, or other metallic elements.^[^ ^62^, ^63^, ^64^ ^]^ Another advantage of Gd‐based biomaterials in bone tissue engineering is their ability to enhance the strength and biocompatibility of other materials while also exhibiting antibacterial and anti‐inflammatory properties, which together enhance bone regeneration (**Figure**. **2**).^[^ ^9^ ^]^

### 3.1 Gadolinium‐Containing Biomaterials Combined with Other REEs Promote Bone Regeneration

Several studies have reported the good osteogenic properties of Gd‐containing biomaterials when combined with other REEs (**Table** **1**).

**Table 1:** Osteogenic properties of gadolinium‐containing biomaterials combined with other rare earth elements.

Gd	Experimental group	Control group	In vitro studies	In vivo studies	Results	Refs.
Gd_2_O_3_	In vitro: OS (0.01, 0.1, 1 µg/mL) Gd_2_O_3_: Eu^3+^ NT suspension; In vivo: Gd_2_O_3_:Eu^3+^ NT suspension (20.0, 100.0, 500.0 mg k^−1^g)	In vitro: OS In vivo: 0.1% HPMC	ALP assay, Western blot, RT‐PCR (BMP‐2, OCN, Runx‐2), micro‐CT (BMD), biomechanics detection (3‐point bending test)	ICR mice, oral gavage	In vitro, increased ALP activity, proliferation and mineralization; in vivo, enhanced osteogenesis‐related gene expression; increased bone mineralization through activation of the BMP signaling pathway; increased BMD and bone trabeculae	[65]
Gd	GDY–Mg alloy (0.5–2.0)	Culture medium	ALP assay	–	GDY1.5 and GDY2.0 increase osteogenesis activity	[66]
Gd^3+^	Sm/Gd‐HAP coating	HAP	ALP assay;	–	Stimulated the differentiation of MC3T3‐E1 cells and facilitated bone formation	[67]

*Abbreviations*: GDY, gadolinium (Gd), dysprosium (Dy), and yttrium; ALP, alkaline phosphatase; RT‐PCR, reverse transcription quantitative polymerase chain reaction; Runx2, Runt‐related transcription factor 2; OS, osteogenic supplements; MTT, methyl thiazolyl tetrazolium; HAP, hydroxyapatite; MC3T3‐E1, preosteoblasts; BMD, bone mineral density; CT, computed tomography; BV/TV, bone volume to total bone volume;HPMC, hydroxylpropyl methyl cellulose; ICR, Institute of Cancer Research; NT, nanotubes; BMP, bone morphogenetic protein.

The favorable characteristics of Gd_2_O_3_ include its homogeneous hollow center, fluorescence, and paramagnetism.^[^ ^34^ ^]^ It is thus often used in combination with europium (Eu), which promotes osteogenesis, angiogenesis, and neurogenesis in addition to exhibiting antimicrobial and antitumor effects.^[^ ^8^ ^]^ For example, Eu‐doped Gd_2_O_3_ nanotubes (NTs) were shown to dose‐dependently enhance the alkaline phosphatase (ALP) activity of murine preosteoblast MC3T3‐E1 cells as well as cell proliferation and mineralization.^[^ ^65^ ^]^ Both real‐time PCR (RT‐PCR) and Western blot (WB) showed that Gd_2_O_3_:Eu^3+^ NTs increase the expression of markers of osteogenesis. Moreover, the Gd_2_O_3_:Eu^3+^ NTs promoted osteogenesis in mice in vivo. The synergy between Gd‐containing biomaterials and Eu can be exploited in composite systems to achieve excellent biological functions in osteogenesis.

The osteogenic effect of a novel GDY–Mg alloy, consisting of Gd, dysprosium (Dy), yttrium (Y), zirconium (Zr), and magnesium (Mg) metals, was evaluated in vitro by Nie et al.^[^ ^66^ ^]^ ALP activity in a MC3T3‐E1 culture system was significantly enhanced by the addition to the culture medium of 0.5–2.0 GDY–Mg alloy. Sathishkumar et al. explored the properties of AISI 316L stainless steel coated with samarium/gadolinium‐doped hydroxyapatite (Sm/Gd‐HAP) in terms of corrosion resistance, protein expression, osteocompatibility, and osteogenic differentiation.^[^ ^67^ ^]^ The Sm/Gd‐HAP‐II coating not only promoted the viability and proliferation of MC3T3‐E1 cells but also regulated their osteogenic differentiation.

These results demonstrate that the combination of Gd‐containing biomaterials with other REEs can further improve the biological functions of Gd‐containing biomaterials.

### 3.2 Gadolinium‐Containing Biomaterials Combined with Metallic Elements Promote Osteogenesis

Metal‐based materials have been well studied with respect to their biological applications, given their good mechanical strength, corrosion resistance, and degradability.^[^ ^68^, ^69^, ^70^ ^]^ They have therefore been assessed when combined with Gd, including for their ability to promote osteogenesis (**Table** **2**).

**Table 2:** Osteogenic properties of gadolinium‐containing biomaterials combined with metallic materials.

Gd	Experimental group	Control group	In vitro studies	In vivo studies	Results	Refs.
Gd	Mg–10Gd discs	Mg disc	ALP assay, RT‐qPCR(ALP, OC;OPN, COL1A1, BMP6);	–	Promising effect on osteoblastogenesis in vitro	[62]
Gd	Zn–0.4Gd alloy	Zn	ALP assay, RT‐PCR(ALP, OCN, COL 1, Runx‐2)	–	Significantly better osteogenesis and osseointegration than with pure Zn.	[72]
Gd^3+^	CS, GdPO_4_/CS, GdPO_4_/CS/Fe_3_O_4_ scaffolds	Blank control	ALP assay, ARS, RT‐PCR(COL‐1, Runx2, BMP‐2, ALP), Western blot	Critical‐sized calvarial‐defect (diameter, 5 mm; thickness, 2 mm) female SD rats (200–250 g)	Activation of the BMP‐2/ RUNX2/ Smad/ pathway by GdPO4‐based scaffolds, with excellent osteoinductivity	[82]
Gd^3+^	Gd‐coated anodized Mg alloy	AZ31 Mg alloy	RT‐PCR (COL‐1, Runx‐2), Bradford assay (OC, OPN)	–	Gd ions released from the coating enhanced proliferation and osteogenesis	[76]
Gd^3+^	MHAP‐ and MHAP/Starch/WST‐coated Ti plates	HAP	ALP assay, ARS;	–	Better induction of osteoblast growth with MHAP/ Starch/ WST	[77]
Gd^3+^	GMNG hydrogel and 6:4 NG hydrogel groups	Blank control	ALP assay, ARS, Western blot (BMP‐2, Runx‐2, OPN),	Tibial defect (Φ 2.5 × 3 mm) in SD rats	After Gd‐TCPP introduction, the GMNG hydrogel effectively promoted new bone formation	[85]

*Abbreviations*: hBMSc, human bone marrow‐derived mesenchymal stem cells; ARS, alizarin red staining; MCS, mesoporous calcium silicate; COL‐1, collagen type1; MHAP, minerals (Mg^2+^ and Gd^3+^) substituted hydroxyapatite; COL‐1, collagen type1; WST, wollastonite; CS, composite scaffold; HUCPVCs, human umbilical cord perivascular cells; RT‐qPCR, semi‐quantitative reverse transcription polymerase chain reaction; OPN, osteopontin; OC, osteocalcin; SD rats, Sprague Dawley rats; Tb Th, trabecular thickness;HPMC, hydroxylpropyl methyl cellulose; Gd‐TCPP, Gd metalated 5,10,15,20‐tetrakis(4‐carboxyphenyl)porphyrin; GMNG hydrogel, MoS2 nanoroses and Gd‐TCPP co‐doped NAGA/Gel‐MA hydrogel; MG63, human osteosarcoma cells; ROBsC, primary rat osteoblasts cell; Tb.N, trabecular number; Tb.Sp, trabecular separation.

For example, the ability of zinc (Zn) to promote osteoclastogenesis and inhibit osteoblast activity has been exploited in orthopedic implants.^[^ ^71^ ^]^ The addition of small amounts of Gd to form Zn–0.4Gd alloys significantly increased the osteogenic and osseointegrative effects of Zn over a limited dose range, with only 0.4 ng elemental Gd/mL detected in the leachate of the alloy.^[^ ^72^ ^]^ A 25% extract of the alloy induced the expression of osteocalcin (OCN) genes.^[^ ^73^ ^]^ In the study of Yang et al., pure Zn and Zn–0.4Gd alloy implants were removed from rat femurs 8 weeks after their implantation. Hematoxylin and eosin (H&E) staining showed a continuous transition zone between the Zn–0.4Gd alloy implants, degradation products, and new bone, with a high degree of bone maturity, no interference from the fibrous layer and, in contrast to the pure Zn group, no evidence of mild inflammation.^[^ ^72^ ^]^ These results showed that the introduction of small amounts of Gd in Zn implants can promote bone regeneration and integration.

Mg has also been applied as an in vivo implant due to its good degradation properties and biocompatibility.^[^ ^74^ ^]^ Combinations of Gd and Mg exhibit good solubility, which increases with temperature.^[^ ^75^ ^]^ Gd‐coated Mg alloys were prepared by anodization and electrophoretic deposition and evaluated for their osteogenic properties. The results of RT‐PCR, ALP assay and Bradford assays consistently showed that Gd^3+^ released from the coatings enhances osteogenesis.^[^ ^76^ ^]^ The surface activity of the implant was modified by the application of mineralized (Mg^2+^ and Gd^3+^) hydroxyapatite (MHAP)/starch/wollastonite (WST), which improved biocompatibility and osteointegration.^[^ ^77^ ^]^ In experiments examining the survival, differentiation, and calcium mineralization of MG‐63 osteoblasts, this novel coating was able to promote bone formation, thus demonstrating its potential in bone regeneration applications.

Costantino et al. have shown that Mg–10Gd modulates the immune response and promotes the differentiation of macrophages in the inflammatory environment toward the M2 pathway, thereby increasing tissue repair.^[^ ^78^ ^]^ Another study found that Mg‐10Gd enhances osteoblast formation in vitro, evidenced by high ALP activity and the significant increase in osteopontin (OPN) gene expression in different culture systems, as determined by RT‐qPCR.^[^ ^79^ ^]^ These results are consistent with the synergistic effects of Mg–10Gd on mineralization during osteoblastogenesis. Furthermore, the ability of Mg–10Gd to mediate macrophage function by regulating the expression of macrophage immunomodulatory factors (TNF α, IL1 β, and IL6/oncostatin M) and osteogenic factor (BMP6), suggested that it promotes bone regeneration through immunomodulatory pathways. Femoral cortex puncture experiments in rats demonstrated the rapid and heterogeneous disintegration of Mg–10Gd after 12 weeks.^[^ ^80^ ^]^ Gd‐containing biomaterials in combination with Mg should thus be evaluated for use in bone tissue engineering in clinical settings.

A study of the properties of orthopedic implants of Mg–1.8 Zn–0.2 Gd alloy in rats described their rapid osseointegration (within the first month) with the surrounding bone, no adverse effects on bone remodeling within the first 2 months, and only a small amount of residue remaining after 6 months.^[^ ^81^ ^]^ The potential of Mg–1.8 Zn–0.2 Gd alloys as orthopedic implant material, under the strict control of the Gd content and Mg and Zn ratios, also merits further investigation.

Zhao and coworkers took advantage of the excellent near‐infrared (NIR) light absorption and photothermal conversion efficiency of Gd to construct a GdPO_4_/CS/Fe_3_O_4_ scaffold, which, under NIR light irradiation increased the local temperature. The scaffold was shown to inhibit tumor growth and promote bone regeneration.^[^ ^82^ ^]^ The Gd^3+^ released by the GdPO_4_ nanorods enhanced angiogenesis and osteogenesis, by activating the BMP‐2/SMAD/RUNX2 pathway. In a further study that based on critical‐sized calvarial defects in rats. The morphometric analyses showed significantly greater bone mineral density (BMD) and bone volume to total bone volume (BV/TV) values in the GdPO_4_/composite scaffold (CS) and GdPO_4_/CS/Fe_3_O_4_ groups than in the control and CS alone groups, which indicating the synergistic effect of Gd combined with Fe in bone tissue regeneration.

Gd methylated 5,10,15,20‐tetrakis(4‐carboxyphenyl)porphyrin (Gd‐TCPP) is a Gd‐based complex with good enhancement of photodynamic therapy and applications in MRI.^[^ ^83^ ^]^ Tian et al. showed that molybdenum Mo^4+^ inhibits osteoclast differentiation by scavenging reactive oxygen species (ROS) and inhibiting mitochondrial biogenesis in osteoclasts.^[^ ^84^ ^]^ In another study, Huang et al. combined MoS_2_, as a photothermal stimulant, with Gd‐TCPP and introduced N‐acryloyl glycinamide (NAGA) and gelatin methacrylate (Gel‐MA), with good biocompatibility and degradation properties, to form MoS_2_ nanoroses and Gd‐TCPP co‐doped with NAGA/gel‐MA hydrogel (GMNG hydrogel).^[^ ^85^ ^]^ ALP, alizarin red S (ARS) staining, and WB showed that Gd^3+^ released from the hydrogel with the highest Gd‐TCPP content had the strongest ability to promote osteogenesis in cultured primary rat osteoblasts after 21 days. The GMNG hydrogel was also implanted in a rat femoral defect model; the micro‐CT results showed a much higher BV/TV and trabecular number (Tb.N) in the GMNG hydrogel group than in the blank group. The reduced separation of trabeculae in the experimental group indicated good osseointegration between newly formed and old bone. The in vitro and in vivo results demonstrated the good osteogenic effect of GMNG hydrogels, and that Gd combined with Mo exerts synergistic effects in bone tissue regeneration.

In summary, the above studies show that the combination of Gd and metallic elements substantially improves the intrinsic properties of metals, enhances the biocompatibility of the materials, and promotes their osteogenic abilities. The doping of Gd in metallic materials may thus be effective in improving their osteogenic capacity.

### 3.3 Gadolinium‐Containing Biomaterials Combined with Nonmetallic Bioactive Materials Promote Bone Regeneration

Nonmetallic bioactive materials have also been widely used in tissue engineering due to their good biocompatibility, their physicochemical similarities to tissues, and their stable binding.^[^ ^86^ ^]^ However, their use in promoting osteogenesis has been limited by their low mechanical strength and rapid degradation in vivo. These issues have been successfully addressed experimentally by doping nonmetallic bioactive materials with Gd (**Table** **3**).^[^ ^87^ ^]^

**Table 3:** Osteogenic properties of gadolinium‐containing biomaterials combined with nonmetallic materials.

Gd	Experimental group	Control group	In vitro studies	In vivo studies	Results	Refs.
Gd^3+^	Gd1/3‐BG scaffolds	Undoped BG scaffolds (84SiO_2_⋅12CaO⋅4P_2_O_5_)	ALP assay, ARS, Western blot, RT‐PCR(OCN, BSP),Akt inhibitor treatment, histological analysis, sequential fluorescent labeling	Critical‐sized cranial bone defect (diameter:5 mm) in male SD rats	In hBMSCs, increased osteogenic differentiation via Akt/gsK3β pathway; in rats, increased bone regeneration	[92]
Gd^3+^	Gd1/5MCS/CTS and Gd1/3MCS/CTS scaffolds	MCS‐CTS scaffolds	Western blot, RT‐PCR (Runx2, ALP, COL‐1), ALP assay, ARS	Critical‐sized cranial bone defect (diameter:5 mm, thickness 2 mm) in male SD rats	In rBMSCs, Gd‐doped scaffolds activated Wnt/β‐catenin signaling pathway to enhance osteogenesis; in rats, both Gd‐doped scaffolds significantly accelerated bone mineralization and new bone deposition	[90]
Gd^3+^	Gd‐WH/CS scaffolds	WH/CS scaffolds	ALP assay, ARS. Western blot, RT‐PCR (OCN, OSX, COL1A1),	Cranial bone defect (diameter: 5 mm) in 12‐week‐old female SD rats	In hADSCs, enhanced osteogenic differentiation via GSK3β/β‐catenin signaling pathway; in rats, increased bone regeneration	[89]
Gd	[Gd@C_82_(OH)_22_]n nanoparticle	physiological saline	ALP assay, ARS; ELISA. oil red O staining, Western blot, immunofluorescence	8‐week‐old female OVX SD rats	Activation of the BMP signaling pathway in MSC differentiation prevented bone loss in rats	[95]
Gd^3+^	GdPO_4_·H_2_O/PLGA; GdPO_4_·H_2_O@SiO_2_/PLGA; GdPO_4_·H_2_O@SiO_2_–APS/PLGA; PBLG‐g‐GdPO_4_·H_2_O/PLGA	PLGA;	ALP assay. ARS, RT‐PCR (ALP, COL‐1, Runx2)	–	NP pronounced mineralization	[91]
Gd_2_O_3_	BG‐Gd_2_O_3_	BG	EDS analysis	–	Gd elements promoted calcium phosphate deposition on the glass surface and decreased glass dissolution	[98]
Gd	Gd@C_82_(OH)_22_ (CM + 10 mM)	growth medium, CM	ALP assay; ARS; oil red O staining; Western blot; RT‐PCR	–	Protection of hBMSC viability in inflammatory microenvironment by reducing ROS; regulation of osteogenic differentiation via the JNK/STAT3 signaling pathway in the inflammatory microenvironm‐ent	[94]

*Abbreviations*: Gd‐BG, gadolinium‐doped bioglass; CTS, chitosan; MCS, mesoporous calcium silicate; rBMSCs, rat bone marrow‐derived mesenchymal stem cells; WST, wollastonite; hADSCs, human adipose‐derived stem cells; Gd‐WH/CS, gadolinium‐doped whitlockite/chitosan; WH‐CS, whitlockite/chitosan; SPP1, secreted phosphoprotein 1; BGLAP, bone gamma‐carboxyglutamate protein; APS, 3‐aminopropyltriethoxysilane; PLGA, poly(lactic‐co‐glycolic acid); PBLG, poly(g‐benzyl‐L‐glutamate); SHEDs, mesenchymal stem cells derived from deciduous teeth; SEM, scanning electron microscopy; EDS, energy‐dispersive X‐ray analysis; ICP‐MS, inductively coupled plasma‐mass spectrometry; OVX, ovariectomized; NAGA, N‐acryloyl glycinamide; Gel‐MA, gelatin methacrylate; ROS, reactive oxygen species; CM, conditioned medium.

Whitlockite (WH, Ca_18_Mg_2_(HPO_4_)_2_(PO_4_)_12_) is an important inorganic constituent of bone tissue that contributes to its continuous supply of Mg^2+^ and PO_4_^3–^, which stimulate membrane ion channels, as shown experimentally using human‐tonsil‐derived mesenchymal stem cells (hTMSCs), thus enhancing their osteogenic activity.^[^ ^88^ ^]^ Xiao et al. used Gd^3+^ and WH to obtain Gd‐doped WH/chitosan composite scaffolds (Gd‐WH/CS). Both RT‐PCR and WB showed that Gd dose‐dependently decreased glycogen synthase kinase 3β (GSK3β) and increased β‐catenin activity. It also increased the expression of osteogenesis‐related genes (OCN, osterix [OSX], and collagen [COL]1A1).^[^ ^89^ ^]^ In a rat critical‐sized bone defect model, the BMD and BV/TV of new bone after 8 weeks were higher in the Gd‐WH/CS group than in the WH/CS group. These findings suggest that the addition of Gd into WH scaffolds enhances their osteogenic effect.

Gd‐MCS (mesoporous calcium silicate)/CTS (chitosan) scaffolds were constructed using the freeze‐drying technique of Liao et al.^[^ ^90^ ^]^ Compared with MCS/CTS scaffolds, Gd‐MCS/CTS scaffolds accelerated the deposition of extracellular matrix and ALP, with the amounts of both increasing with the increasing percentage of doped Gd. Besides, RT‐PCR showed that the Gd‐MCS/CTS scaffolds significantly increased the expression of osteogenesis‐related genes, such as ALP, COL‐1 and RUNX2, while WB showed that Gd‐MCS/CTS scaffolds up‐regulated the level of β‐catenin over that obtained with MCS/CTS scaffolds. These results suggest that Gd‐doped scaffolds enhance bone tissue regeneration by regulating the Wnt/β‐catenin signaling pathway. When tested in a rat cranial defect model, BMD, BV/TV, and Tb.Th were higher in the Gd1/5MCS/CTS and Gd1/3MCS/CTS groups than in the MCS/CTS group. These results demonstrate the positive role of Gd‐containing nonmetallic bioactive scaffolds in promoting osteogenesis.^[^ ^90^ ^]^

The physicochemical features of GdPO_4_‐H_2_O@SiO_2_ have also been characterized. GdPO_4_‐H_2_O@SiO_2_‐APS and PBLG‐g‐GdPO_4_‐H_2_O resulted in stronger T1‐weighted MRI signals, greater ALP activity, significant up‐regulation of COL I and II gene expression, and increased calcium mineralization.^[^ ^91^ ^]^ Thus, Gd in combination with SiO_2_ can promote osteogenesis.

Zhu et al. examined the effect on osteogenesis of Gd1/7‐BG (bioactive glass) (Gd:2.04 wt%), Gd1/5‐BG (Gd:2.68 wt%), and Gd1/3‐BG (Gd: 3.94 wt%) scaffolds. ALP assays showed the better performance of the Gd‐BG scaffolds than the BG scaffolds in vitro.^[^ ^92^ ^]^ Significantly higher expression of the osteogenic genes OCN and BSP (human bone sialoprotein) was observed in human bone marrow mesenchymal stem cells (hBMSCs) associated with Gd‐BG scaffolds than with BG scaffolds. The results of ARS staining were consistent with those of gene expression. The ability of the Akt inhibitor LY294002 to reduce the up‐regulated osteogenic differentiation of Gd1/3‐BG lysate‐treated hBMSCs was demonstrated by WB, ALP assay, and ARS staining. These results implicated the Akt/GSK3β pathway in the stimulatory effect of Gd‐BG on osteogenic differentiation. Moreover, when tested in a rat cranial bone defect model, the Gd1/3‐BG scaffold significantly promoted the formation of new bone at the site of bone defects, as evidenced by the higher BV/TV and BMD values and the increased deposition of new bone tissue. The area of OCN positivity was higher in defects implanted with the Gd1/3‐BG scaffolds than with the BG scaffolds. These results demonstrate the positive role of Gd1/3‐BG scaffolds in promoting osteogenesis.

Gd@C_82_(OH)_22_ possesses immunomodulatory effects and the ability to aggregate in bone tissue.^[^ ^93^ ^]^ Lin et al. used macrophage‐derived conditioned medium to construct an in vitro model of the macrophage‐derived inflammatory microenvironment.^[^ ^94^ ^]^ They found that Gd@C_82_(OH)_22_ reduces bone damage by decreasing ROS levels. WB and RT‐PCR showed that Gd@C_82_(OH)_22_ dose‐dependently regulates stem cell differentiation in the inflammatory microenvironment through the JNK/STAT3 signaling pathway, with low doses of Gd@C_82_(OH)_22_ promoting osteogenesis. Besides, Yang et al. examined [Gd@C_82_(OH)_22_]*_n_* nanoparticles (NPs) and found that they promoted the osteogenic differentiation of MSCs by increasing both ALP activity and mineralized nodule formation.^[^ ^95^ ^]^ RT‐PCR showed that the genes encoding RUNX2, bone morphogenetic protein (BMP)‐2, and ALP were significantly up‐regulated. Based on these results and those of WB, the authors concluded that [Gd@C_82_(OH)_22_]*_n_* NPs activate the BMP signaling pathway to enhance the differentiation of MSCs into osteoblasts. In 3‐month‐old ovariectomized (OVX) rats injected with these NPs at a dose of 228 ug kg^−1^ day^−1^ for one month, there was a significant increase in BMD, BV/TV, and Tb.Th compared with the saline‐treated control group, evidencing the ability of [Gd@C_82_(OH)_22_]*_n_* NPs to increase bone density and prevent osteoporosis.^[^ ^95^ ^]^

In summary, the above studies show that doping with Gd can overcome shortcomings in the mechanical properties of nonmetallic active materials, allowing their use as biomaterials that promote bone regeneration.

Bone defects are accompanied by inflammation, infection, and inflammatory injury. The ROS generated in response may lead to osteoblast death and inhibit the differentiation of BMSCs and precursor osteoblasts. Therefore, the removal of excess ROS is crucial in bone regeneration. In inflammatory environments, Gd‐based biomaterials promote osteogenesis, mainly by removing excess ROS and promoting macrophage M2 differentiation but also through their antimicrobial properties. Besides, they also enhance the proliferation and migration of stem cells, preosteoblasts, and osteoblasts to the site of bone defects. And their subsequent adhesion by the activation of a classical signaling pathway, including Wnt/β‐catenin, BMP‐SMAD, and MAPK, and by increasing the expression of ALP, OPN, OCN, COL‐I, BMP‐2, and RUNX2 in BMSCs.^[^ ^65^, ^72^, ^76^, ^82^, ^85^, ^95^ ^]^ Through its stimulation of the extracellular calcium (Ca^2+^)‐sensing receptor, Gd^3+^ increases intracellular Ca^2+^ levels and thus the mitogenic response, as demonstrated in MC3T3‐E1 cells (Figure 2).^[^ ^96^ ^]^

In conclusion, Gd, whether in combination with other REEs, metallic elements, or nonmetallic bioactive materials, is able to induce osteogenesis, both in vivo and in vitro while improving the mechanical strength and biocompatibility of the material used to repair bone defects. Moreover, it also confers photothermal properties to the material and improves MRI. Aseptic loosening and bacterial infection are the main causes of bone implant failure, so materials that promote osteogenesis need to be antimicrobial while being inherently biocompatible and promoting bone regeneration.^[^ ^97^ ^]^ In addition to the gadolinium‐containing material's inherent good osteogenic properties, its antimicrobial properties significantly contribute to osteogenesis in vivo.

## 4 Gadolinium and Antimicrobial Activity

**Figure 3:**
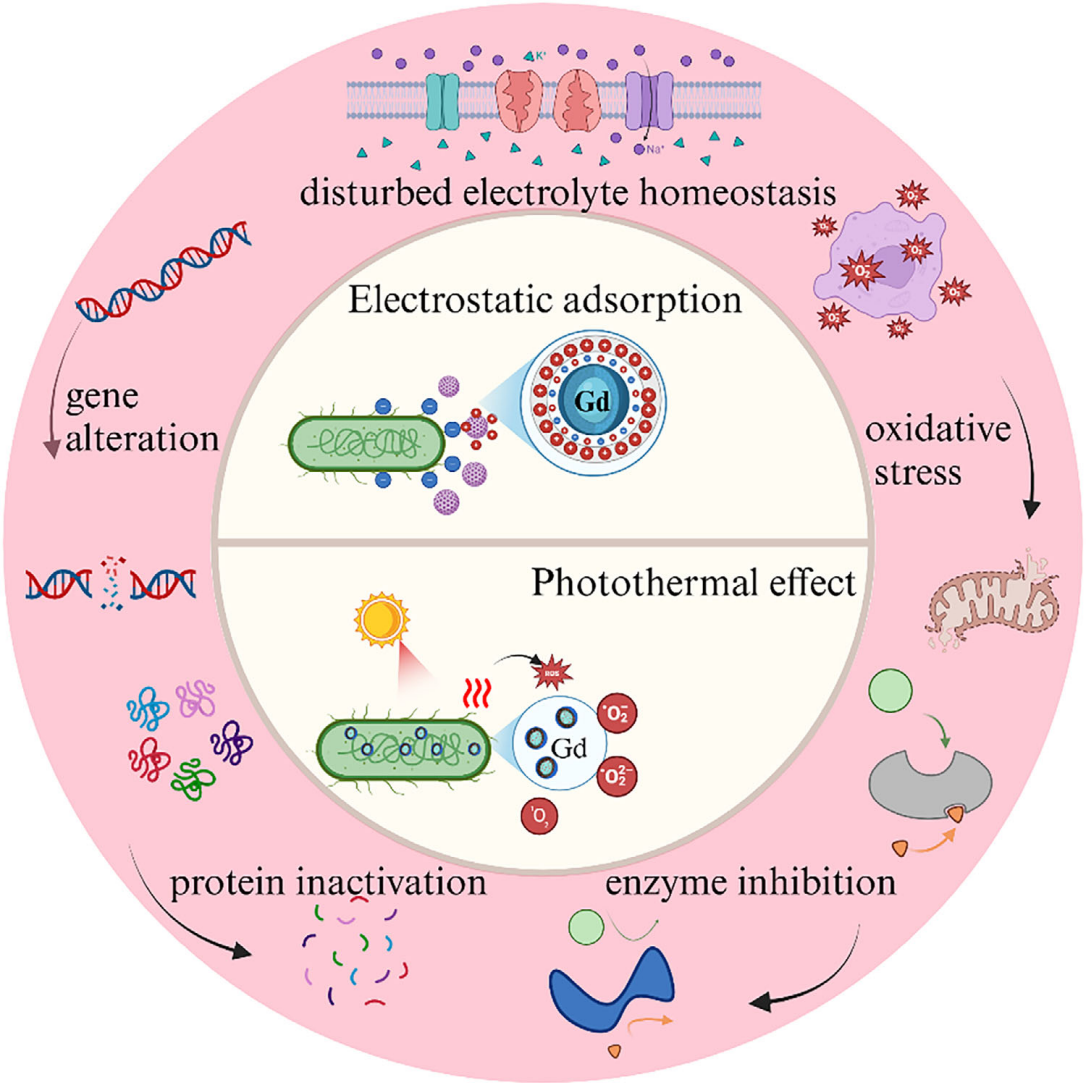
Gadolinium‐containing bioactive materials for antimicrobial applications. Gadolinium‐containing biomaterials antimicrobial by altering the surface charge of the material to enhance the attraction between bacteria and act as photothermal converters for antimicrobial properties.

Similar to other REEs, Gd^3+^ exhibits excellent antimicrobial activity, including through its photodynamic properties, which induce singlet oxygen formation under visible light irradiation as well as the disruption of bacterial DNA (**Figure** **3**; **Table** **4**).

**Table 4:** Gadolinium‐containing biomaterials in antimicrobial applications.

Bacterial species	Experimental group	Control group	Colony size assessment	Results	Refs.
*Escherichia coli, Staphylococcus aureus*	Gd‐coated anodized Mg alloy	AZ31 Mg alloy; bare	Spread plate method (CFU)	Nearly complete (99%) inhibition of bacterial colony formation	[76]
*S. aureus, Bacillus subtilis, E. coli, Pseudomonas aeruginosa*	4‐ASA/ Gd‐CDs	4ASA‐CDs	MIC assay	Excellent antibacterial behavior against *S. aureus* and *E. coli*	[101]
*B. subtilis, Serratia marcescens, E. coli, S. aureus*, and *P. aeruginosa*	Gd_2_O_3_ NP	Amoxicillin	MIC assay	Better antibacterial activity against *B subtilis, S marcescens, E coli*, and *S aureus* than against P*. aeruginosa*; increased activity at higher NP concentrations	[102]
*E. coli, S. aureus*, and *Salmonella typhimurium*	Asc‐Gd_2_O_3_ NP	Kanamycin	MIC assay, spread plate method (CFU)	Better antimicrobial potential against gram‐negative bacteria	[103]
*Pseudomonas fluorescens, S. aureus, Streptococcus enterica, B. subtilis* and *E. coli*	GdVO_4_/FG	FG	Spread plate method (CFU)	Nearly complete (> 96%) reduction in bacterial cell viability in the GdVO_4_/FG groups; in the FG group, 54%, 55%, 50%, 52%, 53% bacterial cell killing in 3 h	[99]
*E. coli, Klebsiella pneumoniae, Pseudomonas syringae*, and *B. subtilis*	Gd:ZnS NP coated with 3% biotin at 1, 5, or 10 µg/ml	Uncoated Gd:ZnS NP, 10 µg/ml	Disc diffusion method	Increased zone of inhibition with increasing NP concentration	[100]

LB, Luria broth; 4‐ASA: 4‐aminosalicylic acid, CD: carbon dots, MIC, minimum inhibitory concentration; Asc, l‐ascorbic acid; NP, nanoparticles.

Shandilya et al. developed GdVO_4_/FG photocatalysts with a large specific surface area by depositing GdVO_4_ onto fluorine‐doped graphene (FG). Under visible light irradiation, GdVO_4_/FG induced the production of ROS, which effectively killed *Pseudomonas fluorescens*, *Staphylococcus aureus*, *Streptococcus enterica*, *Bacillus subtilis*, and *Escherichia coli*.^[^ ^99^ ^]^

Gd‐based biomaterials are often doped with additional substances to modify their surface charge. In a recent study, Gd was incorporated as a doping element into zinc sulfide (ZnS) NPs to create Gd:ZnS NPs, the surfaces of which were modified with biotin to enhance the ability of the NPs to penetrate bacterial cell membranes. The biotin‐modified Gd:ZnS NPs exhibited antimicrobial activity against a wide range of pathogenic bacteria, including via the generation of ROS. Electrostatic interactions between the positively charged NPs and the negatively charged bacterial cell membranes facilitated the adsorption of the NPs onto the bacterial cells and thus their ability to cause cell damage or death.^[^ ^100^ ^]^

In a study of the antimicrobial properties of 4‐ASA/Gd‐CDs based on bacterial counts, determined using the MTT method, the minimum inhibitory concentrations (MICs) against gram‐positive *S. aureus* and *B. subtilis* were 30 and 17 µg/mL, respectively; against gram‐negative *E. coli* and *Pseudomonas aeruginosa*, the MICs were 210 and 35 µg mL^−1^, respectively. The presence of NH_2_ and NH^3+^ groups on the surface of the carbon dots, resulting in their positive surface charge, may have contributed to their antimicrobial activity, as the electrostatic attraction would have induced the leakage of cytoplasmic components and thus bacterial cell death. The larger specific surface area of smaller carbon dots allows for their increased binding to bacterial cells, in turn enhancing their antibacterial effect.^[^ ^101^ ^]^

In another study of the antibacterial properties of Gd_2_O_3_ NPs, using the above four bacterial species, antimicrobial activity was shown to increase with higher concentrations of the NPs, with the strongest inhibition obtained with a concentration of 1 mg mL^−1^. The primary mechanism involved the targeting of the proteins on the surface of the bacterial cell membrane, leading to bacterial cell rupture and death.^[^ ^102^ ^]^

The activity of gadolinium oxide NPs coated with l‐ascorbic acid (Asc) has also been investigated. The MICs of the Asc‐Gd2O3 NPs against *E. coli*, *S*. *aureus*, and *Salmonella typhimurium* were 8, 12, and 10 mg mL^−1^, respectively, comparable to those of kanamycin, used as the control.^[^ ^103^ ^]^ The antimicrobial effects were attributed to the diffusion of the NPs into the cell membrane, leading to oxidative stress, altered cell membrane permeability, disturbed electrolyte balance, enzyme inhibition, protein inactivation, gene alteration, and ultimately cell death.^[^ ^104^ ^]^ Electrostatic interactions generated by the zeta potential of NPs adversely affected exchange pumps and other cell membrane components. Simultaneously, the antioxidant properties of Asc were enhanced by the presence of Gd^3+^ radical ions, which increased the toxicity of the Asc‐Gd2O3 NPs.

In summary, Gd‐containing NPs exhibit potent antibacterial activity by causing the destruction of genetic material, altering enzyme activity, and disrupting cell proteins, and by facilitating binding to the bacterial membrane; in addition, their photothermal properties increase bacterial cell permeability.

## 5 Gadolinium as an Antitumor Agent

**Figure 4:**
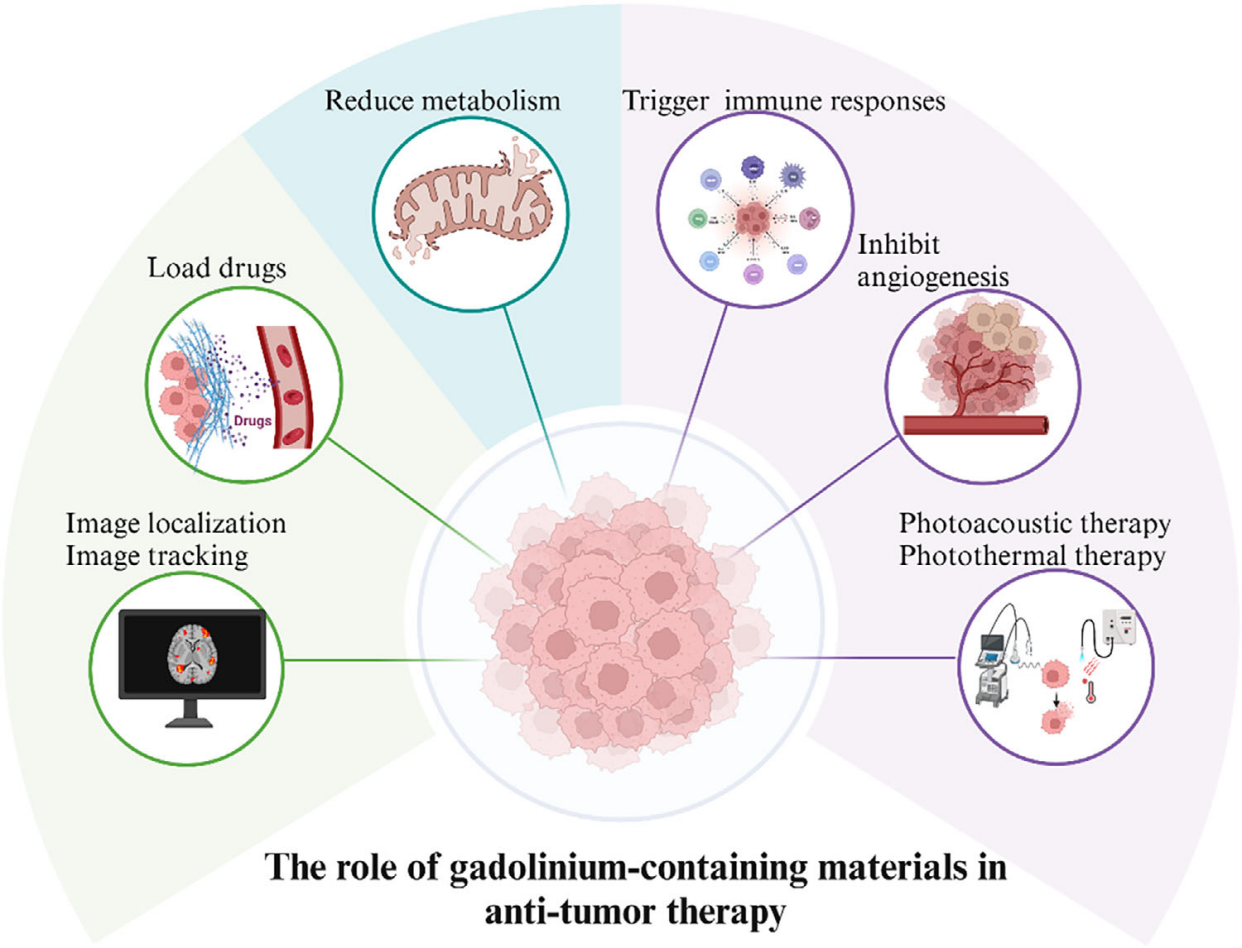
Gadolinium‐containing bioactive materials for antitumor applications. In tumor diagnosis and treatment, gadolinium‐containing biomaterials can play the role of contrast agents, drug carriers, as well as antitumor effects by inducing oxidative stress and inhibiting angiogenesis.

Gadolinium is used as a contrast agent to enhance the visualization of tumor location and contours. It has also been employed as a carrier to deliver drugs to specific sites while enabling tracking and monitoring. However, a large number of studies have examined the use of Gd‐containing biomaterials as an antitumor agent, based on its ability to disrupt mitochondrial function, induce oxidative stress, and impede ATP synthesis on reaching the tumor site.^[^ ^29^, ^30^, ^31^ ^]^ It also exerts a photothermal effect, promotes the maturation of immune cells, activates a systemic antitumor immune response, and damages blood vessels at the tumor site, thereby inhibiting the nutrient supply and inducing the death of tumor cells.^[^ ^105^, ^106^ ^]^ Furthermore, Gd enhances the immune response, by promoting immune cell maturation and activating systemic antitumor immune responses (**Figure** **4**).^[^ ^107^, ^108^ ^]^

In addition to the composition of the material itself, the morphology of the material, such as the groove structure and the surface nanorod array structure that facilitate cell adhesion and antimicrobial action, can have an impact on the biological properties of the material; therefore, in addition to changing the chemical composition of the material, implant materials with excellent antimicrobial capabilities and good cytocompatibility can be designed simply by changing the surface morphology of the material.^[^ ^97^ ^]^ Based on this, researchers have constructed models to better utilize the multiple properties of gadolinium‐containing elements. Tumor Microenvironment‐Induced Degradable Ultrathin Gadolinium Oxide Nanoscroll was constructed by Wu et al. The easy degradation of NSs into small nanofragments in acidic tumor tissues facilitates the penetration accumulation and diffusion of Gd2O3 NSs in deep tumor tissues. diffusion. Meanwhile, Gd2O3 NSs/DOX exhibited pH‐responsive DOX release, with significantly higher DOX release observed at pH 5.0 (35%) and pH 6.5 (33%) compared to pH 7.4 (20%).The NSs/DOX and its degradation product Gd3+ had targeted and significant killing properties on tumor cells with low toxicity to normal cells, and coupled with the MRI contrast Together with MRI contrast, gadolinium oxide nanoscroll exerts targeted contrast and killing effects in antitumor therapy.^[^ ^109^ ^]^

Xia et al. constructed PPF‐Gd NSs based on the ability to efficiently load therapeutic drugs due to the high porosity and 2D morphology of lanthanide‐based porphyrin metal‐organic framework nanosheets (PPF‐NSs) that result in an extraordinary surface area. The excellent photoluminescence and magnetic properties of PPF‐Gd NSs make them very promising carriers for drug delivery and bimodal imaging probes for cancer therapy.^[^ ^110^ ^]^

Gd‐labeled NPs are specifically recognized and taken up by αvβ3 integrin‐positive cells within the tumor vasculature, resulting in enhanced targeting for vascular imaging.^[^ ^111^ ^]^ Gd can also identify areas of inflammatory activity, thereby allowing the assessment of regions with enhanced angiogenesis.^[^ ^112^ ^]^ Additionally, Gd in the form of metal fullerene NPs, Gd@C_82_(OH)_22_, was shown to inhibit tumor angiogenesis by suppressing the expression and activity of matrix metalloproteinases. Gd‐fullerene nanoparticles (GF‐Ala) undergo dimensional swelling and exhibit magnetic properties under radiofrequency irradiation. The resulting size expansion and magneto‐thermal effects alter vascular morphology and induce bleeding, thereby destroying the tumor vasculature and disrupting the nutrient supply to the tumor, in turn inhibiting tumor growth.^[^ ^113^ ^]^

Mitochondria play a crucial role in cellular energy metabolism, the regulation of immune responses, and tumor immunotherapy.^[^ ^114^, ^115^ ^]^ They also support tumor cell activity by supplying essential macromolecules to meet the demands of rapidly proliferating tumor cells. Mitochondrial metabolic dysfunction is a hallmark of tumor cells, characterized by the overproduction of ROS, which can activate the oncogenic signaling pathways that promote tumor cell survival and growth. However, the abnormal mitochondrial metabolism of tumor cells also increases oxidative stress and induces mitochondrial damage, which can be exploited in antitumor therapy. A study investigating the effect of Gd^3+^ on mitochondrial F1FO‐ATPase activated by Mg^2+^ or Ca^2+^ showed that Gd^3+^ binds to and thus alters the activity of F1FO‐ATPase, thereby affecting mitochondrial function. Gd^3+^ also increases the responsiveness of mitochondrial permeability transition pores to Ca^2+^, to directly interfere with Ca^2+^ entry, physically altering membrane structure and permeability.^[^ ^116^ ^]^

In an innovative approach, Zhang et al. developed Gd‐oxide nanosheets that they combined with acid‐responsive polymers to create high‐field MRI probes, ultrasensitive for visualizing submillimeter tumors.^[^ ^117^ ^]^ Gd induces the generation of excess ROS, which cause double‐stranded DNA breaks, inhibit cell proliferation, and thereby enhance the efficacy of radiotherapy.^[^ ^118^ ^]^ Gd radicals also promote immunogenic cell death (ICD) and prevent extracellular ATP synthesis, which together create a pro‐inflammatory tumor microenvironment, induce the maturation of dendritic cells, and contribute to an effective antitumor immune response.^[^ ^119^ ^]^

Similarly, certain compounds containing Gd derivatives can be utilized as photosensitizers and acoustic sensitizers for photodynamic and sonodynamic antitumor therapy. Gd in the form of porphyrin compounds (GdPorP) efficiently generates ROS under ultrasound radiation. The accumulation of ROS in the mitochondria induces large‐scale ICD, including by activating a systemic antitumor immune response.^[^ ^120^ ^]^ Under continuous wave laser irradiation, GdPc generates a single linear oxygen (^1^O_2_) with a quantum yield of up to 0.672, leading to cell death.^[^ ^121^ ^]^ Under pulsed‐wave laser irradiation, GdPc also generates ^1^O_2_ while producing a photoacoustic shock wave through nonradiative relaxation that causes local thermoelastic expansion and mechanical damage to cancer cells, which is particularly effective in the hypoxic tumor microenvironment.

Gd‐doped carbon dots exhibit concentration‐dependent photothermal conversion in the NIR region. The absorbed laser energy is converted to thermal energy under 810‐nm laser irradiation. The high temperatures generated at the tumor site facilitates the selective destruction of cancer cells.^[^ ^122^ ^]^ Similarly, Gd‐doped poly (diisopropanolaminoethyl methacrylate) nanoparticles (GPDPA NPs) demonstrate high photothermal conversion efficiency under NIR laser irradiation. These NPs have been examined for their use in controlled drug release, in response to the acidic pH of tumors and external NIR laser irradiation, such that the drug exposure of normal tissues is minimized.^[^ ^123^ ^]^ Liu et al. incorporated Gd^3+^ into nanostructures to enhance the photothermal conversion efficiency of MAGM NPs.^[^ ^124^ ^]^ MAGM NPs served as a drug carried for the anti‐inflammatory drug meloxicam, to achieve its controlled release, resulting in significant anticancer effects in a 4T1 mouse model of breast cancer. Yuan et al. designed multimetallic organic framework nanosheets carrying doxorubicin (DOX‐Gd‐TCPP) that were irradiated with 660 nm laser light. The conversion of laser energy into thermal energy in combination with photodynamic therapy and the administration of the chemotherapeutic drug DOX resulted in enhanced therapeutic efficacy.^[^ ^125^ ^]^

Gd can also be incorporated into gadolinium metallofullerene (GMF), which is then integrated into pH‐responsive polymer NPs. This system can transition from a hydrophobic to a hydrophilic state in the acidic microenvironment of tumors. GMF can also be used to encapsulate Gd^3^⁺ within fullerene cages, thereby minimizing direct exposure while amplifying MRI signals and, when used a carrier of DOX, simultaneously triggering its release.^[^ ^126^ ^]^ In a similar manner, Gd introduced into magnetic DOX‐loaded NPs (ipGdIO‐Dox) was effective as an MRI contrast agent in response to the weakly acidic tumor microenvironment.^[^ ^127^ ^]^ The release of Fe^2+^ from the particles catalyzed the generation of highly toxic hydroxyl radicals (–OH) from hydrogen peroxide (H_2_O_2_), thereby increasing cellular oxidative stress, damaging mitochondria and cell membranes, and inducing ferroptosis in cancer cells. Gd oxide (GO) NPs and Gd poly (acrylic acid) macrocyclic compounds (GP) have also been loaded into the hollow cores of mesoporous organosilicon NPs in combination with DOX to create composite NPs with anticancer activity. Degradation of the disulfide bonds of the particles resulted in the release of the latter into the glutathione‐rich tumor microenvironment, along with the encapsulated GO or GP and DOX. Surface modification of the NPs with Arg‐Gly‐Asp (RGD) peptides, which specifically recognize αvβ3 integrins on the surface of tumor cells, can be used to actively target the NPs to tumor cells. Both the size and the surface modifications of the NPs influence their penetration and retention within tumor tissues.^[^ ^128^ ^]^

The applications of Gd in the antitumor setting are therefore multifaceted. Gd‐containing materials can be used as contrast agents to monitor tumor sites and blood flow, as drug carriers to carry antitumor drugs to designated sites, to disrupt mitochondrial function within the tumor microenvironment, and to induce oxidative stress. Gd can also be used to destroy the blood vessels of a tumor site directly, by physical methods, or in its role as a photothermal conversion agent.

## 6 Other Biomedical Applications of Gadolinium

**Figure 5:**
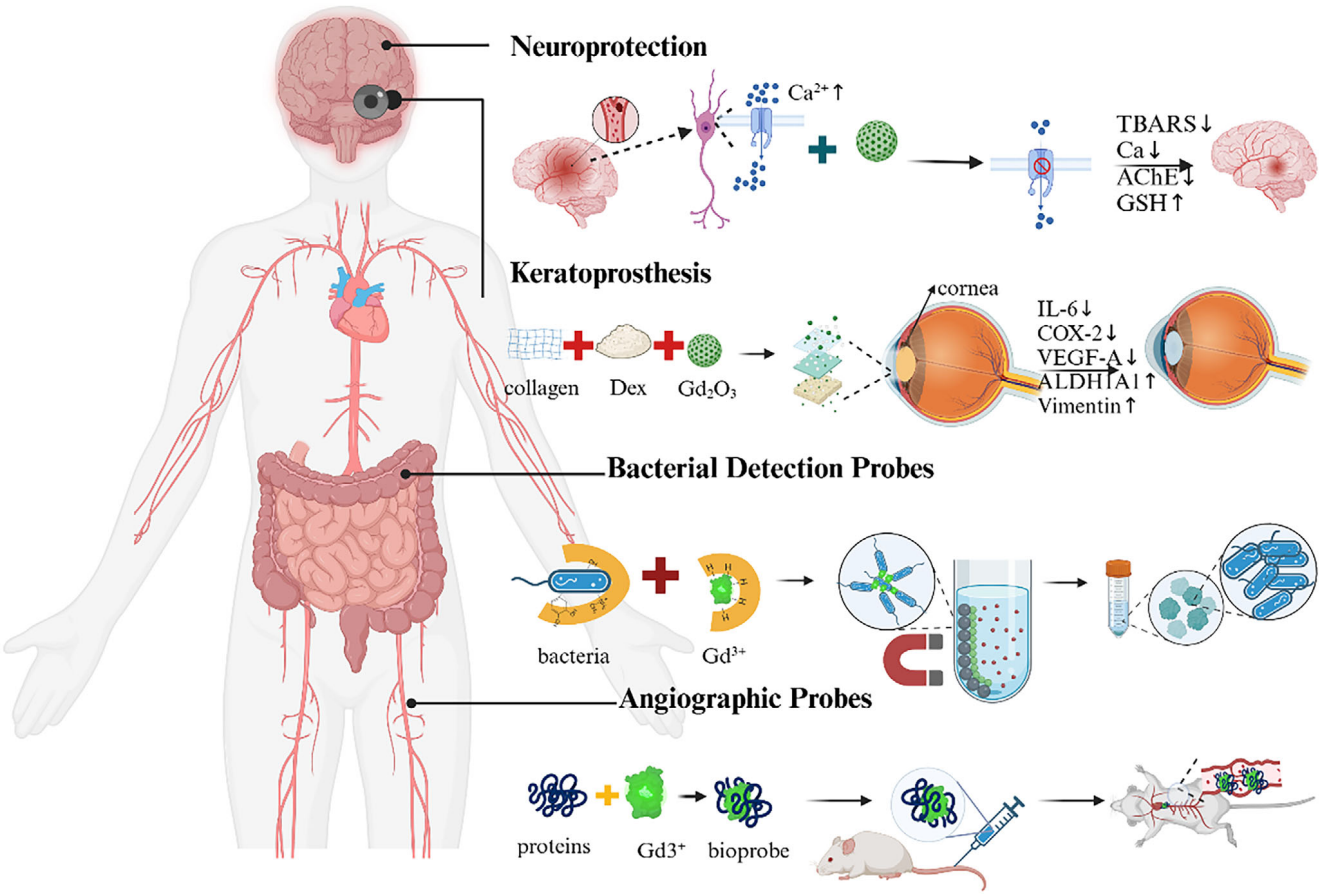
Gadolinium‐containing bioactive materials in other biomedical applications. Gadolinium‐based materials act as calcium channel blockers to inhibit neuroprotection, repair retinal damage by reducing pro‐inflammatory and promoting anti‐inflammatory responses, and act as biological probes for vascular visualization, and bind to bacteria to confer magnetic properties to isolate them.

Other promising applications of Gd‐containing materials include their use as anti‐inflammatory and neuroprotective agents, in corneal repair and regeneration, in bio‐imaging, and as bioprobes for detecting bacteria (**Figure** **5**).

### 6.1 Anti‐Inflammatory Effects

The intravenous injection of GdCl_3_ depletes macrophages through apoptosis, thus reducing macrophage‐mediated inflammation and oxidative stress. This response also involves the decreased expression of HO‐1, ET‐1, and IL‐6 and reductions in the remodeling of the pulmonary vasculature, thereby attenuating pulmonary arterial hypertension.^[^ ^129^ ^]^ In a vascular injury model, Gd^3+^ in combination with alendronate formed a nanosuspension that inhibited restenosis after angioplasty by inhibiting macrophage activity and decreasing the release of the cytokines IL‐1β and TNF‐α secreted by macrophages, in turn reducing circulating monocytes and neointimal formation.^[^ ^130^ ^]^ In patients with rheumatoid arthritis, Gd^3+^ acts as a chemical inhibitor to increase the expression of endoplasmic reticulum stress‐associated proteins, decrease caspase‐3 expression, inhibit transient receptor potential melastatin 7 channel activity, and increase apoptosis of synovial fibroblast‐like cells.^[^ ^131^ ^]^ The combination of Gd‐based and other biomaterials has been used in the early diagnosis and treatment of arthritis. Hes‐Gd_2_(CO_3_)_3_@PDA‐PEG‐DWpeptide NPs were formed by coating Gd_2_ (CO_3_)_3_ NPs with PDA and anchoring a cartilage‐targeting peptide on the surface, followed by loading with naringenin (hesperetin). In addition to good cartilage affinity and MRI applicability, the particles effectively targeted cartilage tissues, inhibited the activation of the NF‐κB/Akt signaling pathway, and protected against IL‐1β‐induced inflammatory responses and apoptosis in chondrocytes following targeted release of the drug in the inflammatory microenvironments.^[^ ^132^ ^]^ Negatively charged Gd‐DTPA2 NPs are repulsed by the glycosaminoglycans (GAGs) in cartilage, such that the distribution of Gd contrast agent is inversely proportional to the amount of GAGs in cartilage. GAGs play an important role in the load distribution and compressive stiffness of cartilage, and their depletion is an early event in the development of osteoarthritis. Therefore, the use of Gd in delayed Gd‐enhanced MRI facilitates the detection of early cartilage degeneration, which is important for the early diagnosis of osteoarthritis and the prevention of its progression.^[^ ^133^ ^]^

### 6.2 Corneal Repair and Regeneration

Gd_2_O_3_ promotes cell regeneration, has anti‐inflammatory properties, and inhibits blood vessel formation such that it holds promise for use as a biocomposite material in the repair and regeneration of corneal cells. Vijayan et al. combined Gd_2_O_3_ NPs with collagen‐dextran composites to form collagen‐dextran‐gadolinium oxide NP (Col+Dex+Gd) hydrogels with anti‐inflammatory and antiangiogenesis properties. The NPs down‐regulated the expression of VEGF‐A and the pro‐inflammatory cytokines IL‐6 and COX‐2 while up‐regulating the expression of ALDH1A1 and vimentin genes, required for corneal cell health and proliferation. In vitro, the NPs accelerated the regeneration and migration of corneal cells to areas of wear.^[^ ^134^ ^]^

### 6.3 Neuroprotective Effect

In ischemia‐reperfusion (I/R) injury, stretch‐activated calcium channels (SACCs) open in response to mechanical forces, leading to massive intracellular calcium accumulation and thus damage to neuronal cells. The use of Gd as a SACC blocker significantly reduced thiobarbituric acid reactive substances, total calcium, and acetylcholinesterase activity while increasing reduced glutathione levels in brain tissue, significantly reducing the volume of cerebral infarcts in I/R‐induced Swiss mice.^[^ ^135^ ^]^ The neuroprotective effects included antioxidant activity and protection against calcium overload. Gd also has a restorative effect on retinal nerve injury. After GdCl_3_ treatment, the expression of pro‐inflammatory factors in the retina was suppressed whereas the expression of anti‐inflammatory factors was up‐regulated, thus inhibiting the overproliferation and activation of M1‐type microglial cells in the retina after optic nerve crush.^[^ ^136^ ^]^ Microglial cells are resident immune cells in the central nervous system. Their hyperactivation after nerve injury leads to secondary damage to retinal ganglion cells. Therefore, GdCl_3_ contributes to the repair and functional recovery of retinal injury by modulating the activity and phenotype of microglial cells, especially after optic nerve injury, by reducing pro‐inflammatory responses and promoting anti‐inflammatory responses.

Alzheimer's disease (AD) is a neurodegenerative disorder that severely affects the quality of life of older adults.^[^ ^137^ ^]^ Aggregation of Aβ peptides, leading to synaptic damage, neuronal death and cognitive decline is a core pathological feature of AD.^[^ ^138^ ^]^ Yin et al. constructed bifunctionalized Gd@C_82_ nanoparticles by introducing hydroxyl and amino groups through a one‐step oxidation reaction to form a Gd@C_82_O_12_(NH_3_)_4_(OH)_17_(NH_2_)_2_NO_2_ structure. f‐Gd@C_82_ NPs significantly reduced Aβ oligomer‐induced neuronal death, inhibited Aβ‐induced ROS generation, and reduced the cell membrane pores that repair Aβ‐induced neuronal morphological damage.^[^ ^139^ ^]^ This design significantly attenuated neuronal toxicity and provided a new strategy for AD treatment.

### 6.4 Gadolinium in Molecular Imaging and as a Biological Probe

Gd is a paramagnetic substance that contains unpaired electrons. By attenuating spin‐spin coherence, Gd contrast agents shorten the T2 relaxation time, observed as a decrease in signal intensity in T2‐weighted imaging. This property explains the extensive use of Gd‐based contrast agents in the imaging of the musculoskeletal, cardiovascular, and central nervoussystems and in oncology.^[^ ^27^, ^140^, ^141^, ^142^, ^143^ ^]^ Moreover, Gd‐containing biomaterials enable dual‐modality imaging, as they can be combined with NIR imaging materials for the noninvasive, high‐resolution imaging of implants or tissue in vivo.^[^ ^144^, ^145^ ^]^

The use of Gd‐containing contrast agents as low‐dose, high‐sensitivity probes and their in vivo degradation have garnered considerable biomedical research attention. In the field of oncology, Gd has been combined with apoptosis‐targeting groups to form tumor‐targeting probes.^[^ ^146^ ^]^ The biocompatibility of Gd‐based imaging nanoprobes has been improved by modifying ultrathin Gd_2_O_3_ nanowire loops to lower their Young's modulus, create spatial repulsion to allow the nonspecific adsorption of proteins, and reduce toxicity.^[^ ^147^ ^]^ Given the abundant carboxyl group (–COOH) on fully carboxylated albumin (BSA‐COOH) and their strong affinity for Gd^3^⁺ in Gd_2_O_3_, BSA‐COOH‐Gd_2_O_3_ nanoprobes with a high metal loading and a high relaxation rate were synthesized. These probes were used to visualize blood vessels with diameters as small as 0.3 and 0.13 mm on MRI at 3.0 and 9.4 T, evidence of the great potential of this approach in imaging.^[^ ^148^ ^]^ In another study, a series of Gd‐based MRI nanoprobes with well‐defined sizes were synthesized. Better tumor accumulation and penetration were observed with the larger nanoprobes A critical size threshold of ≈8.0 nm was determined by evaluating the distribution of the probes in tumors. These findings could help guide the design of nanodrug delivery systems for effective tumor imaging and therapy.^[^ ^149^ ^]^ The construction of nanoprobes containing polydopamine, a high photothermal conversion efficiency material, took advantage of the melting effect of DNA as a linker. When the PDA nanoprobes were subjected to laser irradiation with a photothermal effect, the localized temperature increase caused the denaturation of DNA and thus separation of the contrast agent DTPA/Gd from the nanoprobe such that its chemotherapeutic drugs (e.g., DOX) cargo was released. This mechanism resulted in the precisely controlled release and rapid degradation of the payload in the nanoprobe.^[^ ^150^ ^]^

Gd‐containing materials can also be combined with TPBP, a fluorescent molecule with an aggregation‐induced emission effect, to form a Gd‐DOTA‐TPBP bimodal imaging probe for use in magnetic resonance/fluorescence bimodal imaging, an important tool in early tumor diagnosis.^[^ ^151^ ^]^ In addition, Gd^3+^‐DOTA‐monoamide binds to tropoelastin‐binding peptide (VVGS‐peptide), allowing the more accurate diagnosis of atherosclerosis.^[^ ^152^ ^]^ The peptide in the probe (FFYEGK) self‐assembles into NPs (GFV probe) through π–π stacking. The vancomycin molecule in the probe acts as a targeting ligand that specifically recognizes and binds to the cell wall of *S. aureus* by interacting with the d‐alanine‐d‐alanine dipeptide of the cell wall through hydrogen bonding. This allows specific recognition of infections—and the distinction between inflammation caused by bacterial infections and sterile inflammation—and thus monitoring of the effectiveness of antibiotic treatment.^[^ ^153^ ^]^ Given its high affinity for oxygen‐containing functional groups on the bacterial surface, Gd^3+^ has been chelated with these groups to confer magnetic properties to bacteria. The subsequent application of an external magnetic field results in the rapid magnetic separation of the bacteria. This simple and rapid method has been applied in the detection and isolation of model bacteria such as *S. aureus, E. coli*, and *Fusobacterium sativum* without the complicated preparatory steps needed to functionalize NPs.^[^ ^154^ ^]^ Similarly, the magnetic complex (SA‐PASP‐Gd) was generated by conjugating streptavidin with activated polyaspartic acid via an amide reaction, thereby inducing the strong chelation and adsorption of Gd ions by PASP. By linking the magnetic complex with biotinylated antibodies, a bioprobe for the capture of *Salmonella* in milk was obtained.^[^ ^155^ ^]^

These studies show that Gd‐containing bioactive materials have a wide range of applications in bioimaging and as bioprobes, allowing the sensitive and efficient detection of biomarkers, and in disease diagnosis.

## 7 Conclusion and Perspective

With their excellent biocompatibility, antimicrobial properties, and ability to promote tissue regeneration, REEs have found a wide range of in vivo applications.^[^ ^9^, ^156^ ^]^ Among the REEs, Gd has good paramagnetic properties and is extensively used as a contrast agent in MRI in clinical practice.^[^ ^12^ ^]^ Many studies have demonstrated the excellent function of Gd‐based biomaterials in biomedical settings.^[^ ^90^, ^92^, ^101^, ^157^, ^158^ ^]^ This review examined the abundant research on Gd‐based biomaterials in promoting bone tissue regeneration and as antibacterial and antitumor agents. Gd‐based biomaterials can be combined with other REEs, such as Eu, but also with metallic materials such as Zn and Mg or nonmetallic bioactive materials such as bioactive glass ceramics (BGC) and HAP to form bioactive scaffolds or novel composites. Gd‐based composites retain the properties of the core material, such as biocompatibility and degradability, while also improving its strength and antimicrobial properties. At the same time, Gd significantly enhances osteogenesis, which has raised interest in its use in tissue engineering. However, the antimicrobial properties of Gd are also important. Gd‐containing biomaterials increase the attraction of bacteria by altering their surface charge, thereby increasing bacterial cell membrane permeability and disrupting material exchange, finally resulting in the inactivation of the proteins, genetic alterations, and cell death. The role of Gd in cancer is mainly as a contrast agent that enhances the visualization of tumor location and contour. However, it also acts as a carrier to deliver antitumor drugs to a specific site in the area of the tumor, thereby disrupting mitochondrial function, destroying the vasculature at the tumor site, inducing oxidative stress, and impeding ATP synthesis. The above research indicates that Gd‐containing biomaterials will play an increasing role in the biomedical field in the future.

The limits of this study should be noted. At present, there is no uniform standard for judging the effect of Gd‐containing biomaterials with respect to osteogenesis or their antibacterial and antitumor properties. Articles with positive results have a higher likelihood of being published; therefore, many experiments may have the potential to exaggerate positive results or falsify negative and neutral results for publication. It was not possible to confirm the reliability and accuracy of the results of all the articles included in this study, so some publication bias could not be avoided. Meanwhile, in vivo experiments are indispensable for the articles with the result of “the material has good biological activity”, and the articles in this review were not screened according to whether in vivo experiments were performed or not, so the quality of the articles is somewhat inconsistent.

Inconsistencies in the concentration of Gd‐containing biomaterials, the species of experimental animals, the types of experiments, the experimental period, and the observation criteria hinder comparisons between studies. Since many of the studies were qualitative, a systematic, comprehensive analysis awaits more quantitative data. Furthermore, the in vivo experiments summarized herein were designed for short‐term observations. Further studies are needed to determine whether there is long‐term clinical harm to patients and experimental animals treated with Gd‐containing materials. Determining the appropriate in vivo dosage for the appropriate effect is a direction for future research, which will require more in vivo experiments and longer clinical observation periods. At the same time, how to ensure that the gadolinium‐containing material can be utilized at the lowest possible dosage is also a direction for research. Antitumor is currently an important area of clinical and scientific innovation, and the multipurpose use of one drug is worth studying. For example, in antitumor drugs, how gadolinium‐containing materials can be used as contrast agents while carrying antitumor drugs, and at the same time inhibit angiogenesis, reduce ATP production, and play a role in photothermal efficacy, is a need to be considered while exploring the single properties of the material.

This review summarizes the osteogenic properties of different biomaterials incorporating Gd and their applications as antimicrobial and antitumor agents, with a view to providing a solid base of information for subsequent studies. However, there are still many challenges that must be overcome to maximize the potential of Gd in the clinical context.

## Conflict of Interest

The authors declare no conflict of interest.
